# Retraction: Regulation of Proapoptotic Mammalian ste20–Like Kinase MST2 by the IGF1-Akt Pathway

**DOI:** 10.1371/journal.pone.0289329

**Published:** 2023-07-25

**Authors:** The PLOS ONE Editors

The Moffitt Cancer Center investigated this article [1] and determined that there was evidence of data fabrication and/or falsification in the following figures:

- Fig 1a, AKT panel
- Fig 2a, IB: α-HA IgG panel
- Fig 3b, MST2 panel
- Fig 4c, MST2 panel and Cleaved-MST2 panel

Following their findings of misconduct the Moffitt Cancer Centre recommended retraction of [1].

The PLOS Editors also noted that the Fig 1a and Fig 1c MST2 Cleaved-MST2 panels appear to partially overlap when one of the panels is flipped horizontally. PLOS did not follow up on this additional concern.

The authors did not respond to editorial communications regarding the above concerns and did not provide any underlying data.

In light of the outcome of the institutional investigation and the concerns affecting multiple figure panels that question the integrity of these data, the *PLOS ONE* Editors retract this article.

DK and ZY agreed with the retraction. SS, MDC, SK, and JQC either did not respond directly or could not be reached. ZY stands by the article’s findings.
